# Supplementary material for: MetaRibo-Seq measures translation in microbiomes
Source: Nat Commun. 2020 Jun 29;11:3268. doi: 10.1038/s41467-020-17081-z (PMC7324362; doi:10.1038/s41467-020-17081-z)
Supplement: Supplementary file 10 — Supplementary Data 7 [file 41467_2020_17081_MOESM10_ESM.zip › File2/Confidence_High_Taxonomy/37583_out.krona.html]

Javascript must be enabled to view this page.

members
magnitude
magnitudeUnassigned
count
unassigned
taxon
rank

37583\_out

11

2
11
superkingdom

phylum
1239
11

class
11
186801

order
186802
11


SRS018623\_contig\_number\_contig-100\_35157.35158SRS019068\_contig\_number\_contig-100\_2438.336613SRS020328\_contig\_number\_contig-100\_15600.150012SRS024132\_contig\_number\_contig-100\_13429.43174SRS042966\_contig\_number\_contig-100\_2904.83578SRS049995\_contig\_number\_contig-100\_9390.123748SRS063985\_contig\_number\_contig-100\_19984.101678SRS064276\_contig\_number\_23117SRS077392\_contig\_number\_contig-100\_26069.105138SRS142503\_contig\_number\_contig-100\_23506.67056SRS147271\_contig\_number\_contig-100\_37276.37277
species
11
1898207
